# Correlation Between IVIM-DWI and DCE-MRI Parameters in Soft Tissue Tumors: A Comparative Analysis of Benign and Malignant Lesions

**DOI:** 10.3390/tomography12070099

**Published:** 2026-07-01

**Authors:** Ahmet Peker, Yunus Emre Senturk, Enes Muhammed Canturk, Mohammed Salman Shazeeb

**Affiliations:** 1 Department of Radiology, Koç University Hospital, Istanbul 34010, Turkey; 2 Department of Radiology, University of Massachusetts Chan Medical School, Worcester, MA 01655, USA

**Keywords:** soft tissue tumors, intravoxel incoherent motion imaging, dynamic contrast-enhanced MRI, tumor perfusion

## Abstract

**Objective**: The objective of this study was to investigate the relationship between intravoxel incoherent motion diffusion-weighted imaging (IVIM-DWI) and dynamic contrast-enhanced MRI (DCE-MRI) parameters in soft tissue tumors (STTs). **Methods**: This retrospective study included patients with histopathologically confirmed STTs who underwent both DCE-MRI and IVIM-DWI between March 2022 and February 2024. Patients with prior therapy and lipomatous tumors were excluded. DCE-MRI parameters (K_trans_, K_ep_, V_e_, iAUC) were obtained from pharmacokinetic maps using manually placed regions of interest (ROIs) in the most perfused tumor areas, avoiding necrotic and cystic regions. Corresponding ROIs were applied to IVIM-DWI maps. IVIM parameters (D, D*, f) were calculated using 11 b-values. **Results**: Twenty-nine patients (mean age, 56 ± 18 years; 14 malignant, 15 benign) were included. Interobserver agreement was excellent for DCE-MRI parameters, whereas IVIM-DWI parameters showed moderate-to-good agreement, with D showing the lowest reproducibility. In malignant tumors, f demonstrated strong positive correlations with K_trans_ (r = 0.81, *p* < 0.001) and iAUC (r = 0.79, *p* < 0.001), both of which remained significant after correction for multiple comparisons. fD* was higher in malignant than in benign lesions in the unadjusted group comparison; however, diagnostic performance was not evaluated in the present study. No significant differences were observed for DCE-MRI parameters between benign and malignant tumors. **Conclusions**: IVIM-DWI parameters demonstrated associations with DCE-MRI metrics in malignant STTs and may provide complementary information regarding tumor perfusion. However, the findings should be interpreted cautiously because ROI analysis was limited to a single representative slice. Further validation using larger cohorts and volumetric tumor assessment is required.

## Simple Summary

Soft tissue tumors are often difficult to characterize using conventional magnetic resonance imaging alone. Dynamic contrast-enhanced magnetic resonance imaging is commonly used to evaluate tumor perfusion but requires intravenous contrast administration. This study investigated whether intravoxel incoherent motion imaging, a contrast-free magnetic resonance imaging technique, shows relationships with established perfusion parameters in soft tissue tumors. The results demonstrated meaningful associations between the two techniques, particularly in malignant tumors. Selected IVIM-DWI parameters demonstrated associations with established DCE-MRI perfusion metrics, particularly in malignant tumors. However, these findings should be considered exploratory given the small and heterogeneous cohort, and further studies are needed to validate their potential role in non-contrast perfusion assessment.

## 1. Introduction

The diagnosis of soft tissue tumors (STTs) is often challenging due to their rarity and the substantial overlap in imaging findings between benign and malignant lesions [1]. Although depth, size, and heterogeneity on conventional magnetic resonance imaging (MRI) are widely used parameters for differentiation, their sensitivity, specificity, and consistency remain suboptimal [2,3]. Therefore, advanced MRI techniques such as dynamic contrast-enhanced MRI (DCE-MRI) and diffusion-weighted imaging (DWI) have been increasingly investigated to overcome these limitations [4,5].

The meanings, advantages, and limitations of DCE-MRI parameters are well established in oncologic imaging [6,7]. When the Tofts pharmacokinetic model is applied to DCE-MRI, three primary quantitative parameters are obtained: the extravascular extracellular volume fraction (V_e_), the rate constant (K_ep_), and the transfer constant (K_trans_). Additionally, the semi-quantitative parameter of the initial area under the time–signal intensity curve (iAUC) can be derived [8]. Although results vary across studies, apparent diffusion coefficient (ADC), K_trans_, and iAUC have frequently been reported as useful parameters for differentiating benign from malignant STTs [7]. Beyond lesion characterization, DCE-MRI is also used to select an appropriate biopsy site, assess treatment response, and predict prognosis [9]. It has also been reported to be valuable in postoperative surveillance, particularly in differentiating residual tumor from granulation tissue [10].

Intravoxel incoherent motion (IVIM), a multi–b-value DWI technique, enables estimation of tissue perfusion without contrast administration and therefore represents a promising non-invasive alternative [11,12]. Although introduced in 1986, widespread clinical use was delayed until gradient technology became sufficiently advanced [11,13]. IVIM has been applied to several organs and tumors, including the breast and prostate; however, there is still no consensus regarding the optimal number or selection of b-values, or the most appropriate fitting algorithm [14]. To address this heterogeneity and improve methodological standardization, the International Society for Magnetic Resonance in Medicine (ISMRM) organized an IVIM workshop, which subsequently led to consensus recommendations regarding IVIM acquisition and analysis strategies [15]. The main IVIM parameters include the true diffusion coefficient (D), pseudodiffusion coefficient (D*), perfusion fraction (f), and the composite perfusion marker fD*, which reflects local microcirculation. However, the relative unfamiliarity of these parameters, compared with well-established DCE-MRI perfusion metrics, limits their interpretation and hinders integration into routine clinical practice.

Despite growing interest in IVIM-DWI, the relationship between IVIM-derived perfusion parameters and established DCE-MRI metrics in STTs remains unexplored. Previous studies investigating this relationship have been limited by heterogeneous patient populations, small sample sizes, and incomplete histopathological confirmation [4,5]. Furthermore, potential differences in diffusion–perfusion relationships between benign and malignant tumors have not been adequately explored. In addition, the relationship between the composite IVIM parameter fD* and quantitative DCE-MRI metrics has received limited attention.

Therefore, the aim of this study was to investigate the relationship between quantitative DCE-MRI and IVIM-DWI parameters in histopathologically confirmed STTs. We separately analyzed benign and malignant lesions and evaluated the associations between multiple quantitative DCE-MRI metrics and both conventional IVIM-derived parameters and the composite parameter fD*. Differences between benign and malignant tumors were explored as a secondary analysis. We hypothesized that perfusion-related IVIM parameters, including f, D, and fD*, would demonstrate associations with quantitative DCE-MRI metrics reflecting tumor perfusion. Comparisons between benign and malignant tumors and subgroup-specific analyses were considered exploratory.

## 2. Material and Methods

### 2.1. Study Population

This retrospective study was approved by the Institutional Ethics Committee (2025.398.IRB2.181). MRI examinations included in this study were acquired between March 2022 and February 2024 as part of the routine clinical care. Consecutive patients with histopathologically confirmed STTs who underwent both DCE-MRI and IVIM-DWI were retrospectively screened for eligibility. Written informed consent was waived by the Institutional Review Board.

Patients with a history of chemotherapy or radiotherapy prior to MRI, cases with suboptimal image quality, and patients with lipomatous tumors were excluded. Lipomatous tissue demonstrates distinct biological and imaging characteristics compared with other soft tissue tumors. Fat-containing soft-tissue masses are considered a separate diagnostic group, with specific imaging and management strategies. Moreover, perfusion-based MRI techniques—particularly DCE-MRI—have shown limited diagnostic utility in lipomatous lesions due to their inherently low vascularity and variable enhancement patterns [16,17,18]. Therefore, lipomatous tumors were excluded from this study to avoid potential bias in perfusion analysis.

**Figure 1:**
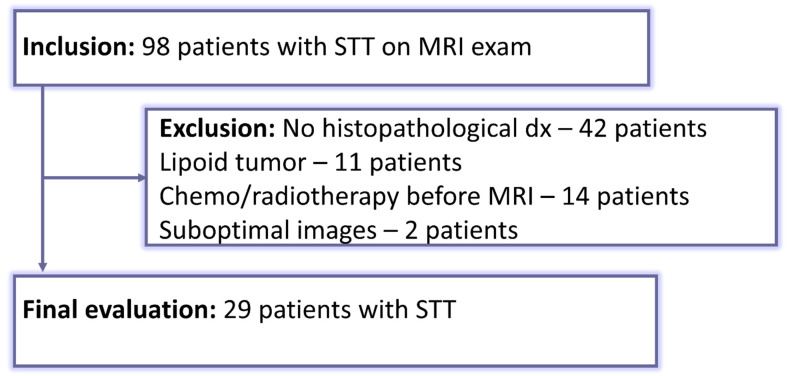
Flowchart illustrating the patient selection process. Dx: diagnosis; STT: soft tissue tumour.

Histopathological confirmation was obtained by biopsy and/or surgical pathology. In all included patients, histopathological diagnosis was established within two weeks after MRI examination. No recurrent tumors were included in the study cohort. Maximum tumor diameter was measured on MRI using the largest lesion dimension identified on multiplanar imaging. After applying the exclusion criteria, 29 patients were included in the final analysis (Figure 1). Anatomical tumor distribution and tumor depth (superficial versus deep) are summarized in Table 1.

### 2.2. Imaging Protocol

All MRI examinations were performed using a 3T system (Skyra, Siemens Healthcare, Erlangen, Germany). The imaging protocol was conducted in a standardized chronological order. First, conventional MRI sequences of the affected extremity were obtained, including axial and/or coronal T1-weighted and fluid-sensitive sequences, according to routine clinical protocols. Subsequently, IVIM-DWI was performed prior to contrast administration using a single-shot spin-echo echo-planar imaging (EPI) sequence in the axial plane with diffusion encoding in three orthogonal directions. Acquisition parameters were as follows: repetition time/echo time (TR/TE), 7000/55 ms; field of view (FOV) adjusted according to lesion size and anatomical location; acquisition matrix, 120 × 134; acquired voxel size, 1.6 × 1.6 × 5 mm; slice thickness, 5 mm; parallel imaging with GRAPPA factor 2; receiver bandwidth, 2194 Hz/pixel; and fat suppression using spectral adiabatic inversion recovery (SPAIR). A total of 11 b-values (0, 20, 40, 80, 110, 140, 170, 200, 300, 500, and 1000 s/mm^2^) were used. One signal average was acquired for all b-values except b = 1000 s/mm^2^, for which two signal averages were obtained. Two-dimensional geometric distortion correction was enabled. The total acquisition time was 3 min 20 s.

Following IVIM-DWI, DCE-MRI was performed. Prior to contrast administration, axial T1-weighted volumetric interpolated breath-hold examination (VIBE) images were acquired. Subsequently, gadobutrol (Gadovist; 0.1 mmol/kg) was intravenously administered using a power injector at a rate of 3 mL/s, followed by a 15 mL saline flush. Dynamic imaging was performed in the axial plane using a spoiled gradient-echo T1-weighted sequence with the following parameters: TR/TE, 5.08/1.77 ms; FOV, 260 × 192 mm; slice thickness, 3.5 mm; flip angle, 15°; temporal resolution, 7 s; and total acquisition time, 255 s.

### 2.3. Image Analysis

All DWI and DCE-MRI datasets were transferred to a dedicated workstation (Syngo.via VB60, Siemens Healthcare, Erlangen, Germany) for post-processing. Parametric maps of K_trans_, K_ep_, V_e_, iAUC, and ADC were generated using the vendor-provided pharmacokinetic modeling tools based on a population-based arterial input function (AIF). Color-coded parametric maps were automatically generated by the software.

All examinations were independently evaluated by two musculoskeletal radiologists (Reader 1: 4 years of experience; Reader 2: 3 years of experience), who were blinded to clinical and histopathological results. DCE-MRI and IVIM-DWI images were analyzed side-by-side on dual monitors.

**Figure 2:**
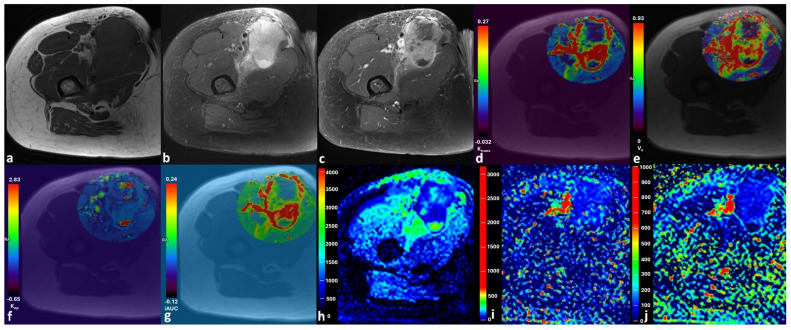
Multiparametric MRI of a 35-year-old woman with a malignant soft tissue tumor located in the anterior thigh. Histopathological examination confirmed CIC-rearranged sarcoma. Images include (**a**) axial T1-weighted image, (**b**) axial proton density–weighted image, (**c**) axial contrast-enhanced fat-suppressed T1-weighted image, (**d**) K_trans_ map, (**e**) V_e_ map, (**f**) K_ep_ map, (**g**) iAUC map, (**h**) D (true diffusion coefficient) map, (**i**) D* (pseudo-diffusion coefficient) map, and (**j**) f (perfusion fraction) map. The figure illustrates the multiparametric assessment of tumor perfusion and diffusion characteristics using both DCE-MRI and IVIM-DWI. RA: right anterior.

Regions of interest (ROIs) were manually placed on a single representative axial slice in areas demonstrating the highest visually assessed enhancement on DCE-MRI, while carefully avoiding necrotic, hemorrhagic, and cystic regions, and large vessels. To ensure spatial correspondence, ROIs were initially defined on DCE-MRI parametric maps and then manually replicated on the corresponding IVIM-DWI maps by matching anatomical landmarks. Given differences in slice thickness and in-plane resolution between sequences, exact voxel-wise matching was not feasible; therefore, ROI placement was performed with careful visual alignment (Figure 2).

**Figure 3:**
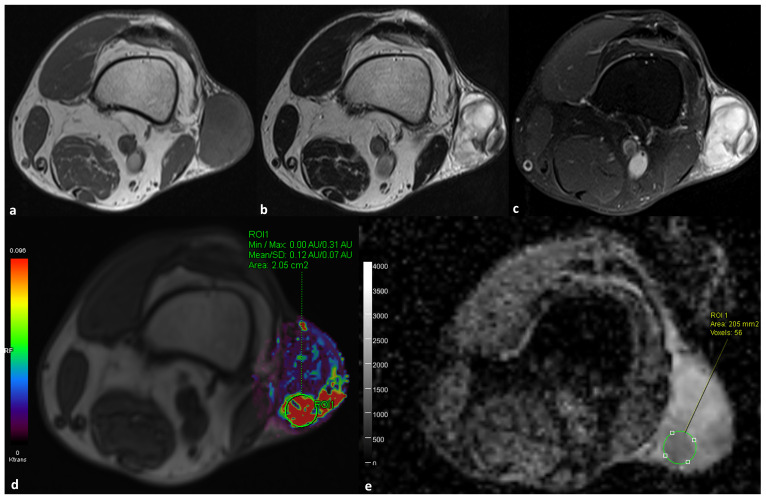
Multiparametric MRI of an 82-year-old man with a malignant soft tissue tumor located at the medial aspect of the knee. Histopathological examination confirmed pleomorphic sarcoma. Images include (**a**) axial T1-weighted image, (**b**) axial T2-weighted image, (**c**) axial contrast-enhanced fat-suppressed T1-weighted image, (**d**) K_trans_ map, and (**e**) D map (true diffusion coefficient). Identical-sized regions of interest (ROIs) were placed at corresponding anatomical locations within the most visually enhancing solid portion of the tumor while avoiding necrotic and cystic areas. The figure demonstrates the ROI-matching strategy used for quantitative comparison between DCE-MRI and IVIM-DWI parameters. RF: right foot.

This ROI-based approach was chosen to target the most biologically active tumor regions, although it may not fully reflect intratumoral heterogeneity and may introduce selection bias (Figure 3).

To assess measurement reproducibility, all ROI placements and measurements were repeated after a 6-week interval in a randomized order. Both interobserver and intraobserver agreement metrics were evaluated. For subsequent statistical analyses, the mean values of the measurements obtained by the two readers were used.

### 2.4. DCE MRI Calculation

DCE-MRI data were processed using a dedicated workstation (Syngo.via VB60, Siemens Healthineers, Erlangen, Germany). Perfusion parameters, including K_trans_, K_ep_, V_e_, and iAUC, were calculated using the Extended Tofts pharmacokinetic model. A population-based Parker-type AIF automatically implemented by the software was used for all DCE-MRI analyses, without manual arterial selection. The plasma volume fraction (v_p_) was not available as an output parameter in the vendor-provided software and was therefore not included in the analysis.

A fixed baseline T1 value of 1000 ms and contrast agent relaxivity (r_1_ = 4.5 L·mmol^−1^·s^−1^) were used for all pharmacokinetic calculations. Quantitative perfusion parameters (K_trans_, K_ep_, and V_e_) were derived using the Extended Tofts model and a population-based intermediate-flow arterial input function implemented by the vendor-provided software. The use of a fixed baseline T1 value and a population-based AIF may have influenced parameter accuracy.

Motion correction was automatically applied by the software using a reference dynamic frame, and its adequacy was visually verified in all cases. Temporal smoothing was selectively applied when motion-related signal fluctuations were observed on time–intensity curves, based on visual assessment.

The initial area under the gadolinium concentration–time curve (iAUC, IAUGC60) was calculated from the gadolinium concentration–time curve generated by the software. All analyses were performed using standardized software settings to ensure consistency across patients, although differences in acquisition protocols may limit direct comparability with other studies.

### 2.5. IVIM Calculations

IVIM analysis was performed using dedicated diffusion post-processing software (Frontiers DWI, Version 8.5; Siemens Healthineers, Erlangen, Germany). A segmented bi-exponential fitting approach was applied. First, the true diffusion coefficient (D) was estimated using a linear fit of the logarithmic signal decay at higher b-values (≥200 s/mm^2^). Subsequently, the perfusion fraction (f) and pseudo-diffusion coefficient (D*) were calculated using nonlinear fitting of the full set of b-values.

A total of 11 b-values (0–1000 s/mm^2^) were used for IVIM modeling, with multiple low b-values included to improve estimation of perfusion-related parameters. The combined perfusion parameter (fD*) was automatically derived as the product of f and D*.

Given the known susceptibility of IVIM parameters, particularly D*, to noise and fitting instability, a standardized fitting approach was applied across all patients to improve consistency.

To ensure spatial correspondence, IVIM parameters were extracted from ROIs that were initially defined on DCE-MRI maps and then manually replicated on IVIM-DWI images using anatomical landmarks. This ROI-based approach enabled direct comparison between techniques but may not fully capture intratumoral heterogeneity.

### 2.6. Statistical Analysis

Statistical analyses were performed using IBM SPSS Statistics (version 26; Armonk, NY, USA). The normality of data distribution was assessed using the Shapiro–Wilk test. Group comparisons were performed with the Mann–Whitney U test, while correlations between imaging parameters were evaluated using Spearman’s rank correlation coefficient. To account for multiple comparisons in correlation analyses, *p*-values were adjusted using the Benjamini–Hochberg false discovery rate (FDR) correction. All correlation analyses across the overall cohort, benign subgroup, and malignant subgroup were treated as a single family of tests, resulting in 48 correlations in total. FDR correction was not applied to the exploratory between-group comparisons. Continuous variables are presented as mean ± standard deviation (SD) to facilitate comparison with previous studies, although nonparametric statistical tests were used for group comparisons and correlation analyses. No a priori sample size calculation was performed because this was a retrospective exploratory study based on the available eligible cohort.

Interobserver and intraobserver agreement for quantitative measurements was assessed using the intraclass correlation coefficient (ICC), based on a two-way random effects model with absolute agreement. A *p*-value less than 0.05 was considered statistically significant.

## 3. Results

### 3.1. Patient Characteristics

A total of 29 patients (10 females and 19 males) were included in the study. Fourteen patients were diagnosed with soft tissue sarcoma (STS), and the remaining 15 had benign STTs. The mean age of the patients was 56.0 ± 18.2 years (range, 10–83 years), and the mean lesion diameter (LD) was 61.2 ± 62.9 mm (range, 5–280 mm). The demographic characteristics, anatomical tumor distribution, tumor depth, and histopathological diagnoses are summarized in Table 1.

### 3.2. Interobserver and Intraobserver Agreement

Interobserver and intraobserver agreement levels for all quantitative imaging parameters were assessed using the ICC. Excellent agreement was observed for the DCE-MRI parameters, with interobserver ICC values ranging from 0.90 to 0.96 and intraobserver ICC values ranging from 0.92 to 0.95. Specifically, the interobserver and intraobserver ICC values were 0.93 and 0.94 for K_trans_, 0.90 and 0.92 for K_ep_, 0.96 and 0.95 for V_e_, and 0.94 and 0.93 for iAUC, respectively.

For the IVIM-DWI parameters, interobserver agreement ranged from good to excellent (ICC = 0.66–0.89), while intraobserver agreement showed a similar pattern (ICC = 0.69–0.88). The interobserver and intraobserver ICC values were 0.78 and 0.76 for f, 0.89 and 0.88 for D, 0.66 and 0.69 for D*, and 0.72 and 0.70 for fD*, respectively.

### 3.3. DCE-MRI and IVIM Results of the Tumors

When evaluated separately, the IVIM-DWI parameter fD* was higher in malignant tumors than in benign tumors (321.5 ± 195.2 vs 181.1 ± 117.2 ×10^−3^ mm^2^/s, *p* = 0.029) in the unadjusted exploratory group comparison. Among the DCE-MRI parameters, the mean K_ep_ value was higher in malignant tumors (0.84 ± 0.79 min^−1^) than in benign tumors (0.29 ± 0.17 min^−1^), although the difference did not reach statistical significance (*p* = 0.055). No other significant differences were observed between the two groups for the remaining parameters. Detailed comparisons are summarized in Table 2.

### 3.4. DCE-MRI and IVIM Correlation

When the entire study population was analyzed, a weak yet statistically significant positive correlation was observed between D* and several DCE-MRI parameters. Specifically, D* showed a positive correlation with K_trans_ (r = 0.44, *p* = 0.018), K_ep_ (r = 0.38, *p* = 0.042), and iAUC (r = 0.42, *p* = 0.023). However, none of these associations remained statistically significant (Table 3) after FDR correction (adjusted *p*-values: 0.123, 0.224, and 0.138, respectively).

In benign tumors, nominally significant correlations were observed between D and K_trans_ (r = 0.621, *p* = 0.013) and between D and iAUC (r = 0.615, *p* = 0.015). However, these associations did not remain statistically significant (Table 4) after FDR correction (adjusted *p* = 0.123 for both).

In malignant tumors, strong positive correlations were observed between the perfusion fraction (f) and several DCE-MRI parameters. Specifically, f correlated strongly with K_trans_ (r = 0.81, *p* = 0.001), K_ep_ (r = 0.63, *p* = 0.016), and iAUC (r = 0.79, *p* = 0.001). Additionally, D* showed a significant positive correlation with K_ep_ (r = 0.73, *p* = 0.017). These findings are summarized in Table 4.

After FDR correction for multiple comparisons, only the correlations between f and K_trans_ and between f and iAUC in malignant tumors remained statistically significant (adjusted *p* = 0.024 for both).

## 4. Discussion

In this study, distinct patterns of correlation between IVIM-DWI and DCE-MRI parameters were observed in STTs, both in the overall cohort and within benign and malignant subgroups. In the entire cohort, weak but significant correlations were identified between D* and DCE-MRI parameters (K_trans_, K_ep_, and iAUC). When analyzed separately, significant associations between the IVIM perfusion fraction (f) and selected DCE-MRI metrics were observed in malignant tumors, whereas fewer significant correlations were identified in benign tumors. After correction for multiple comparisons, only the correlations between f and Ktrans and between f and iAUC in malignant tumors remained statistically significant. Several other associations lost statistical significance after FDR correction and should therefore be considered exploratory. The observed associations between f and DCE-MRI parameters may reflect partially overlapping aspects of tumor microvascularity, although these parameters do not represent identical physiological processes. Given the limited sample size, cohort heterogeneity, and multiple testing burden, the remaining findings should be regarded as hypothesis-generating and require further validation.

Our findings are consistent with prior studies. A preclinical study using an orthotopic murine model of rhabdomyosarcoma demonstrated positive correlations between IVIM parameters (D*, f, and fD*) and microvessel density (MVD), as well as associations with pericyte coverage index (PCI) [19]. Similarly, Xiangwen Li et al. reported correlations between IVIM parameters and HIF-1α expression in soft tissue sarcomas, suggesting a relationship between IVIM metrics and tumor hypoxia and angiogenesis [20]. The significant associations observed in malignant tumors in our cohort may reflect partially overlapping aspects of tumor microvascularity. However, because no direct correlation with histopathological markers such as microvessel density, vascular density, necrosis, or tumor grade was performed, any relationship with tumor angiogenesis remains speculative and should be considered hypothesis-generating.

An apparent discrepancy in our findings was that fD* was higher in malignant tumors, whereas no significant correlations were observed between fD* and DCE-MRI parameters. Several factors may explain this observation. First, fD* incorporates D*, a parameter known to have lower reproducibility and greater sensitivity to noise than other IVIM-derived metrics. Second, because fD* is a composite parameter derived from both f and D*, measurement variability from each component may be propagated and amplified. Third, nonlinear fitting of IVIM data may introduce additional instability, particularly for D*-related measurements. Finally, DCE-MRI and IVIM-DWI characterize different aspects of tumor vascular physiology, and residual spatial mismatch between the techniques may have further reduced observed correlations. Therefore, the higher fD* values observed in malignant tumors should be interpreted cautiously and regarded as an exploratory finding requiring validation in larger cohorts.

Compared with previous studies, our results highlight the potential importance of histopathological stratification when evaluating diffusion–perfusion relationships. Marzi et al. reported no significant correlation between IVIM and DCE-MRI parameters [5], which may be explained by differences in tumor composition, inclusion of adipocytic lesions, and the use of semi-quantitative DCE metrics. In contrast, studies such as that by Sun et al. demonstrated moderate correlations between IVIM-derived parameters and DCE-MRI metrics in rectal cancer [21], supporting the notion that diffusion–perfusion relationships may be context-dependent.

Our study is among the limited number of investigations directly comparing IVIM-DWI and DCE-MRI in musculoskeletal tumors. Previous studies have reported variable results regarding the diagnostic performance of individual parameters, including K_trans_, K_ep_, V_e_, iAUC, and D [4,22]. These discrepancies may be attributable to differences in patient populations, imaging protocols, and tumor histology, particularly the inclusion of highly vascular benign tumors.

From a clinical perspective, IVIM-DWI may provide additional perfusion-related information without the need for contrast administration. However, the present findings should be interpreted as exploratory and should not be considered evidence that IVIM-DWI can replace DCE-MRI. Rather, IVIM-DWI should be viewed as a potentially complementary technique, and further studies evaluating reproducibility, diagnostic performance, and clinical outcomes are required before any clinical substitution could be considered.

This study has several important limitations. First, the relatively small sample size and heterogeneous tumor population may limit statistical power and generalizability. In particular, combining multiple tumor subtypes with different vascular characteristics may have influenced correlation results. Second, the use of single-slice ROI-based analysis may not fully capture intratumoral heterogeneity and may introduce selection bias. Furthermore, IVIM-DWI measurements were obtained from the same tumor regions that demonstrated the highest visual enhancement on DCE-MRI in order to ensure anatomical correspondence between techniques. Although this approach minimized spatial mismatch, it may have increased the apparent associations between perfusion-related parameters and should therefore be considered when interpreting the observed correlations. Furthermore, because IVIM-DWI measurements were guided by DCE-MRI-defined tumor regions, the present results do not establish that IVIM-DWI can independently assess tumor perfusion. In addition, differences in slice thickness and spatial resolution between DCE-MRI and IVIM-DWI may have introduced residual spatial mismatch despite careful anatomical alignment. Third, IVIM-derived parameters—especially D*—are known to be sensitive to noise and fitting instability, which may affect reproducibility. Finally, DCE-MRI analysis was based on a population-based AIF and a fixed baseline T1 value, which may have affected the accuracy of the derived pharmacokinetic parameters. Additionally, lipomatous tumors were excluded from the study cohort. Therefore, the present findings may not be generalizable to lipomatous soft tissue tumors, which often demonstrate distinct biological and imaging characteristics. Overall, the present findings should be considered exploratory and require validation in larger, prospective, and more homogeneous cohorts.

## 5. Conclusions

In conclusion, IVIM-DWI parameters showed moderate to strong associations with DCE-MRI metrics in malignant STTs, particularly for perfusion-related parameters. These results suggest that IVIM-DWI may provide complementary information regarding tumor perfusion. However, due to methodological limitations, including single-slice ROI analysis focused on the most visually enhancing tumor regions, cohort heterogeneity, and the exclusion of lipomatous tumors, further validation in larger and more homogeneous populations is required before clinical implementation.

## Figures and Tables

**Table 1:** Demographic and Histopathologic Characteristics of Benign and Malignant Tumors.

	Benign	Malignant	*p*-Value
**Age**	52.6 (±19.6)	59.7 (±15.4)	0.265
**Gender (M/F)**	8/7	11/3	0.245
**Maximum tumor diameter, mm (mean ± SD)**	20.7 (±11.0)	104.6 (±66.9)	<0.001
**Tumor depth**			
Superficial	6	3	
Deep	9	11	
Tumor location			
Upper extremity	8	2	
Lower extremity	3	7	
Trunk/Pelvis	4	5	
**Histopathologic Dx**	Schwannoma (3)	Synovial sarcoma (4)	
	Tendon sheath tumor	Myxofibrosarcoma (2)	
	Superficial angiomyxoma	Leiomyosarcoma	
	Nodular fasciitis	Extra-neuraxial hemangioblastoma	
	Fibroma	Undifferentiated pleomorphic sarcoma (2)	
	Myxoid neurothecoma	Myxoid chondrosarcoma	
	Hemangioma (2)	CIC-rearranged sarcoma (2)	
	Cellular intramuscular myxoma (2)	Spindle cell sarcoma	
	Glomus (2)		
	Low-grade spindle cell proliferation		

SD: standard deviation. Numbers in parentheses indicate the number of cases.

**Table 2:** Comparison of quantitative DCE-MRI and IVIM-DWI parameters between benign and malignant STTs.

Parameters	Benign STTs (*n* = 15)	Malignant STTs (*n* = 14)	*p*-Value
K_trans_ (min^−1^), mean ± SD	0.107 ± 0.114	0.245 ± 0.269	0.097
K_ep_ (min^−1^), mean ± SD	0.296 ± 0.172	0.840 ± 0.791	0.055
V_e_ (%), mean ± SD	0.366 ± 0.333	0.320 ± 0.193	0.965
iAUC (a.u.), mean ± SD	0.149 ± 0.127	0.338 ± 0.362	0.890
f, mean ± SD	0.187 ± 0.155	0.135 ± 0.117	0.570
D (×10^−3^ mm^2^/s), mean ± SD	1.540 ± 0.522	1.429 ± 0.653	0.600
D* (×10^−3^ mm^2^/s), mean ± SD	48.140 ± 33.236	42.064 ± 33.794	0.760
fD* (×10^−3^ mm^2^/s), mean ± SD	181.107 ± 117.244	321.514 ± 195.232	**0.029**

K_trans_: volume transfer constant; K_ep_: rate constant; V_e_: extracellular extravascular space volume fraction; iAUC: area under the curve; f: perfusion fraction; D: true diffusion coefficient; D*: pseudo-diffusion coefficient; fD*: perfusion-related diffusion component. *p* < 0.05 was considered statistically significant.

**Table 3:** Correlation Between IVIM-DWI and DCE-MRI Parameters in the Entire Study Population (*n* = 29).

DCE-MRI Parameter	IVIM Parameter	f	D	D*	fD*
**K_trans_**	Correlation (r)	0.289	0.214	**0.435**	−0.035
	*p*-value	0.129	0.265	**0.018**	0.855
	FDR-adjusted *p*-value	0.367	0.557	0.123	0.924
**K_ep_**	Correlation (r)	0.288	0.181	**0.380**	−0.027
	*p*-value	0.130	0.348	**0.042**	0.891
	FDR-adjusted *p*-value	0.367	0.635	0.224	0.924
**V_e_**	Correlation (r)	0.077	0.202	0.229	−0.043
	*p*-value	0.690	0.293	0.232	0.824
	FDR-adjusted *p*-value	0.901	0.563	0.542	0.924
**iAUC**	Correlation (r)	0.256	0.177	**0.420**	0.026
	*p*-value	0.179	0.357	**0.023**	0.894
	FDR-adjusted *p*-value	0.477	0.635	0.138	0.924

Correlation coefficients (r), *p*-values, and FDR-adjusted *p*-values between DCE-MRI and IVIM-DWI quantitative parameters. Abbreviations: IVIM = intravoxel incoherent motion; DCE-MRI = dynamic contrast-enhanced MRI; f = perfusion fraction; D = true diffusion coefficient; D* = pseudodiffusion coefficient; fD* = flow-related diffusion component. FDR-adjusted *p*-values were calculated using the Benjamini–Hochberg procedure across all 48 correlation analyses. Bold values indicate statistical significance after FDR correction (adjusted *p* < 0.05).

**Table 4:** Correlation Between IVIM-DWI and DCE-MRI Parameters in Benign and Malignant Soft Tissue Tumors.

DCE Parameter	IVIM Parameter	Benign Tumors (*n* = 15) r	*p*-Value	FDR-Adjusted *p*-Value	Malignant Tumors (*n* = 14) r	*p*-Value	FDR-Adjusted *p*-Value
**K_trans_**	**f**	0.096	0.732	0.901	**0.811**	**<0.001**	**0.024**
	**D**	**0.621**	**0.013**	0.123	−0.051	0.864	0.924
	D*	0.461	0.084	0.288	0.508	0.064	0.281
	fD*	−0.186	0.508	0.787	−0.218	0.455	0.753
**K_ep_**	**f**	0.232	0.405	0.694	**0.631**	**0.016**	0.123
	**D**	0.471	0.076	0.281	0.209	0.474	0.758
	D*	0.325	0.237	0.542	**0.626**	**0.017**	0.123
	fD*	−0.086	0.761	0.913	−0.319	0.267	0.557
**V_e_**	**f**	−0.096	0.732	0.901	0.350	0.220	0.542
	**D**	0.482	0.069	0.281	−0.154	0.599	0.879
	D*	0.293	0.289	0.563	0.035	0.905	0.924
	fD*	−0.021	0.940	0.940	−0.106	0.719	0.901
**iAUC**	**f**	0.041	0.884	0.924	**0.792**	**<0.001**	**0.024**
	**D**	**0.615**	**0.015**	0.123	−0.152	0.604	0.879
	D*	0.422	0.117	0.367	0.495	0.072	0.281
	fD*	−0.105	0.708	0.901	−0.119	0.686	0.901

Abbreviations: IVIM = intravoxel incoherent motion; DCE-MRI = dynamic contrast-enhanced MRI; f = perfusion fraction; D = true diffusion coefficient; D* = pseudodiffusion coefficient; fD* = perfusion-related diffusion component. FDR-adjusted *p*-values were calculated using the Benjamini–Hochberg procedure across all 48 correlation analyses. Bold values indicate statistical significance after FDR correction (adjusted *p* < 0.05).

## Data Availability

The data that support the findings of this study are not publicly available due to restrictions imposed by the institutional ethics committee to protect patient confidentiality. De-identified data may be made available from the corresponding author upon reasonable request and with permission of the ethics committee.
